# Supplementary material for: Acyl-Coa Thioesterases: A Rheostat That Controls Activated Fatty Acids Modulates Dengue Virus Serotype 2 Replication
Source: Viruses. 2022 Jan 25;14(2):240. doi: 10.3390/v14020240 (PMC8875275; doi:10.3390/v14020240)
Supplement: Supplementary file 1 [file viruses-14-00240-s001.zip › viruses-1529118-supplementary.pdf]

Table S1. siRNA and oligonucleotide resources used in this study.

**siRNA resources**		
Reagent	Source	Product Identifier
SMARTPool ACOT1 siRNA	Horizon Discovery/Dharmacon	M-034967-00
MISSION® esiRNA human ACOT2	Sigma-Aldrich	EHU104751-20UG
MISSION® esiRNA human ACOT7	Sigma-Aldrich	EHU112971-20UG
**Oligonucleotides**		
	Forward Primer (5'-3')	Reverse Primer (5'-3')
ACOT1/2	AGAGGAAGAGTTGGGCAGAG	TTCGTCCCAGCAGCAGCG
ACOT2	GCCCGAGAGGATGTCTAACA	TCAGGCTCCATTGGTACAGC
ACOT4	AGGAG GGTACAAGAACCCCA	GAGGCTCGATGTAATGCCCA
ACOT6	AGCCGTGGACTTTATGCTGC	AGTACAGTGGCTGTGATGCC
ACOT7	CTGCACCCTGCACGGCTTTG	CGGAAGCTGTGACGATGTTG
ACOT8	GCTCTCGCATTCATAGAGCAT	AAGTTCAGTGGCCATGTTAGC
ACOT9	AAGTTCAGTGGCCATGTTAGC	AATGCCGGCCCTTTATTTTCA
ACOT11	AATCACCAGGGCAACACCTT	CAATGGCCTTCAGCGTAGGG
ACOT12	ACGCTATCGGGGAGCTATTG	TTGCTGTCACTCAGGGATGC
ACOT13	CTCTTCGCCCTTTGTGTCCT	GAGTAATCTTTCCCAAAACTCTCTC

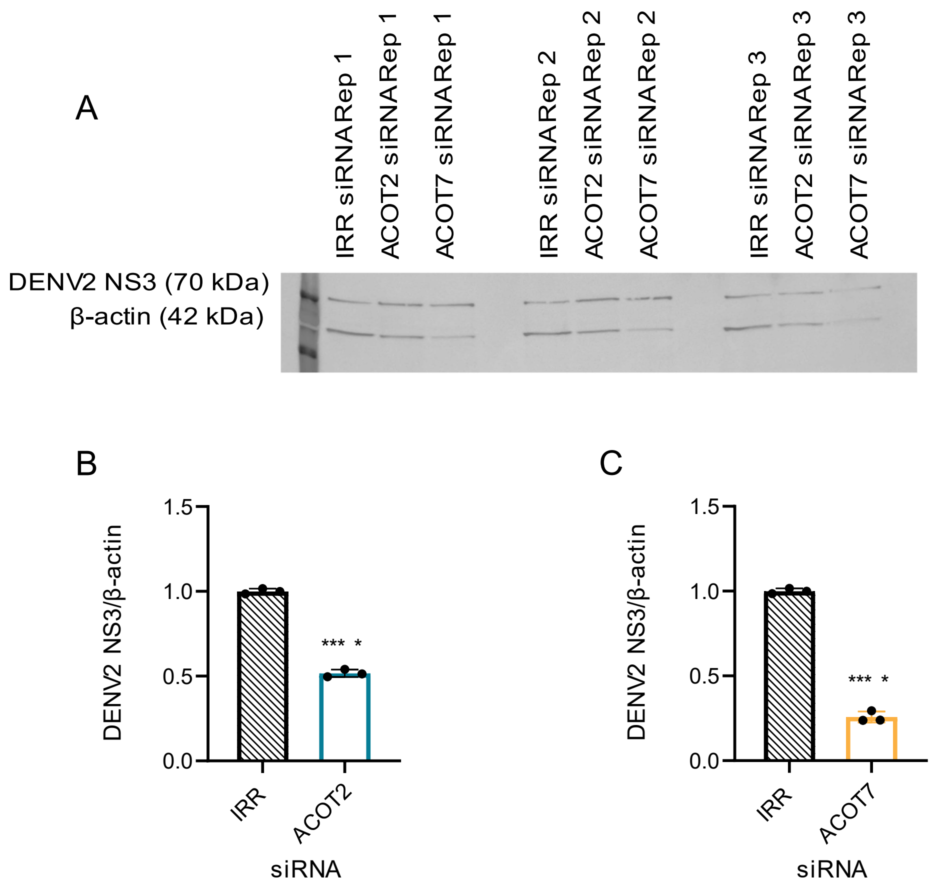

Figure S1 – Loss of function of mitochondrial ACOTs inhibits viral protein translation. Huh7 cells where transfected with either ACOT2, ACOT7, or an IRR siRNA, and then subsequently infected with DENV2 (MOI = 0.3) for 24hr. (A) Cell lysates were prepared and analyzed via western blot. Samples were probed for DENV2 nonstructural protein 3, and β-actin (for normalization). Li-cor IRDyes were used as secondary antibodies. (B-C) and fluorescence intensity of each band was analyzed using area under the curve analysis in ImageJ.
